# Recycled Fibers for Sustainable Hybrid Fiber Cement Based Material: A Review

**DOI:** 10.3390/ma14092408

**Published:** 2021-05-05

**Authors:** Ana Balea, Elena Fuente, M. Concepcion Monte, Angeles Blanco, Carlos Negro

**Affiliations:** Department of Chemical Engineering and Materials, Universidad Complutense de Madrid, Av. Complutense s/n, 28040 Madrid, Spain; anabalea@ucm.es (A.B.); cmonte@ucm.es (M.C.M.); ablanco@ucm.es (A.B.); cnegro@ucm.es (C.N.)

**Keywords:** cement-based materials, fiber reinforced composites, hybrid fiber cement, hybrid composites, recycled fibers, circular economy, sustainability

## Abstract

Reinforcing fibers have been widely used to improve physical and mechanical properties of cement-based materials. Most fiber reinforced composites (FRC) involve the use of a single type of fiber to improve cement properties, such as strength or ductility. To additionally improve other parameters, hybridization is required. Another key challenge, in the construction industry, is the implementation of green and sustainable strategies based on reducing raw materials consumption, designing novel structures with enhanced properties and low weight, and developing low environmental impact processes. Different recycled fibers have been used as raw materials to promote circular economy processes and new business opportunities in the cement-based sector. The valuable use of recycled fibers in hybrid FRC has already been proven and they improve both product quality and sustainability, but the generated knowledge is fragmented. This is the first review analyzing the use of recycled fibers in hybrid FRC and the hybridization effect on mechanical properties and workability of FRC. The paper compiles the best results and the optimal combinations of recycled fibers for hybrid FRC to identify key insights and gaps that may define future research to open new application fields for recycled hybrid FRC.

## 1. Introduction

Cement-based materials have been used in structural applications for many decades even though they have low tensile strength, ductility and crack resistance. The incorporation of steel bars or the addition of dispersed fibers are the most popular reinforcing strategies to overcome the brittleness of concrete. Steel, glass, synthetic polymeric fibers and natural-based fibers are the main reinforcing fibers used in cement-based materials to increase ductility, flexural and tensile strength and to avoid crack propagation [1,2].

Many researchers have studied the effects of different types of fibers and their combination (hybrid composites) on workability, mechanical properties and shrinkage of cement-based materials [3,4,5,6,7,8,9]. In fact, one of the most common hybridization strategies is combining polymeric with cellulosic fibers to improve cement hardening in non-structural fiber reinforced composites (FRC) to replace asbestos [2,9,10,11,12], which provides additional energy saving benefits by avoiding autoclave requirements [13]. Ahmed et al. (2003) [14] proved that the combination of 1% steel fibers with 1.5% polyvinyl alcohol (PVA) fibers, as reinforcement agents of concrete, provided the highest flexural strength with high deflection. Chen and Liu [3] evaluated the effect of three types of fibers (carbon, steel and polypropylene (PP)), in single and dual additions, on the properties of lightweight concrete (LWC). For single fiber addition, carbon and steel fibers can both increase compressive and split tensile strengths, whereas PP fibers decrease these mechanical properties. However, strength increased for all hybrid composites, among which the combination of carbon and steel fibers provided the highest compressive and split tensile strengths, with an increment of 28% and 38%, respectively. Moreover, all hybrid composites showed better restraint effects than the conditions of adding any single type of fiber [3]. The positive effects of hybrid fibers on mechanical properties are attributed to the fact that different sizes and types of fibers offer differing restraints. Therefore, it is accepted that hybridization, by the combination of different fibers, has a synergic effect on FRC mechanical properties [3,15], which has been a hot research topic during the last 20 years.

On the other hand, recycling, waste valorization and circular economy concepts have been widely developed in this period, through strong research efforts aimed to improve the sustainability of FRC production, use and management (at the end of its life) [16,17]. These works consider different strategies: (i) using biodegradable fibers, e.g., cellulosic fibers from wood and from annual plants or lignocellulosic wastes [11,18,19], but the main drawback can be their poor durability in alkaline cement [20], because of that, efforts have carried out on modifying cellulosic fibers [21], use of alternative cement matrix [11,22,23] or pozzolanic additives [16,24,25] to increase chemical resistance of fibers or reduce alkalinity of matrix; (ii) use of nanocelluloses [26,27,28]; (iii) improving the production process [29,30]; and (iv) the use of recycled reinforcing fibers [31,32] and recycled aggregates [33].

Many studies have focused on using recycled reinforcing fibers from recycled plastics, end-of-life-tires and construction waste. Recycling the waste to produce new materials, like concrete or mortar, has been raised as one of the best solutions, due to economic and ecological advantages as well as energy saving in their disposal [34,35]. Therefore, the interest of using recycled fibers (mainly steel and polymeric fibers) in FRC is increasing, as shown in recent reviews [36,37,38,39,40,41,42,43,44,45]. The most promising approach to improve reinforced concrete composites is hybridization [7] and although many studies have been carried out using recycled fibers, the generated knowledge is fragmented.

## 2. Research Significance

In 2016, the amount of generated construction and demolition waste in Europe was around 374 million tons [46]. The European Topic Centre on Waste and Materials in a Green Economy proposed several key actions to apply circular economy concepts across the different stages of building’s lifecycle. Among other actions, they highlight that “the materials are highly durability and therefore have a long lifetime” and “the materials have a high recycled content”. However, the use of a high proportion of recycled fibers in FRC is often limited by the effect on the mechanical properties and durability of the composite. Hybridization has been considered to achieve both increasing recycled fibers use and keeping the required FRC mechanical properties by many different researchers, as proven by the numerous published works. However, in our best knowledge, there is not any specific review about the use of recycled fibers in hybrid FRC; this is the first one. Therefore, the aim of this conceptual paper, developed for the Special Issue, is to review extant knowledge on hybrid cement-based materials containing recycled fibers in its structure, in order to identify key insights as well as gaps that may define future research.

More than 120 works were reviewed and classified into three categories depending on the nature of used fibers. This review analyzes the properties of recycled fibers of different natures and their effect on mechanical properties of hybrid FRC and neatly summarizes the best results obtained on each reviewed work and assesses the optimal combinations of fibers for hybrid FRC containing recycled fibers.

## 3. Data Collection Procedure

The information used in this paper has been collected from SciFinder Scholar, Web of Science and Google Scholar databases and from the previous experience of the authors in this field. These are three databases with wider data coverage, and they complement each other. The largest free database is Google Scholar. It contains nearly 400 million documents comprising articles, patents and citations [47]. However, it is not the best option for structured query and filters are limited. SciFinder Scholar offers algorithmic interpretation of natural language queries for text entries. It covers over 47 million records from more than 50,000 journals from more than 180 countries. It is always actualized because article records are added to SciFinder within 7 days of publication [48]. Both SciFinder Scholar and Web of Science allow one to find reliable, integrated and multidisciplinary research [49], but the research articles included at Web of Science have been strictly evaluated, and this assures that only the most influential, relevant and credible information is included. Furthermore, the works referenced by each article have been collected when they were relevant for this review.

## 4. Recycled Fiber Reinforced Composites (R-FRC)

### 4.1. Type of Recycled Fibers

**Figure 1:**
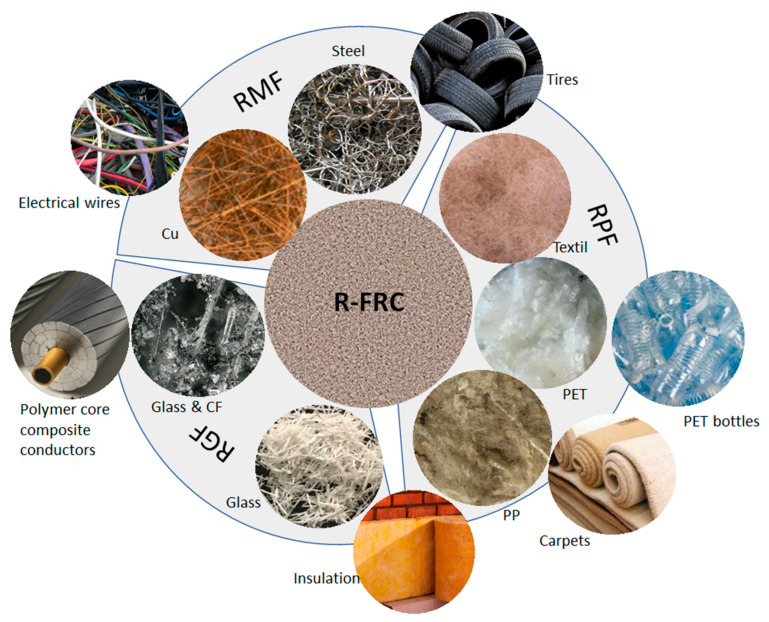
Types of recycled fibers used in R-FRC: recycled metallic fibers (RMF), recycled glass fibers (RGF), carbon fibers (CF) and recycled synthetic polymeric fibers (RPF).

A wide range of recycled fibers have been studied for R-FRC formulation, as shown in Figure 1: recycled metallic fibers (RMF), recycled glass fibers (RGF), carbon fibers (CF) and recycled synthetic polymeric fibers (RPF).

The use of steel fibers is common in hybrid formulations. Waste tires is the most used source to produce recycled-tire steel fibers (RTSF), but other sources such as waste metal lathes, have also been studied with promising results [50]. RTSF are extracted from waste tires by a mechanical recycling process in which the rubber is shredded and granulated, the steel is removed by magnets and the textile is separated by a vacuum [38]. RTSF obtained by this process have irregular shape, with different lengths and diameters, and high flexural strength [38]. In general terms, the type of waste tire (e.g., cars, trucks) is crucial to define the diameter of the fibers, meanwhile the recycling procedure is a key factor to define their length. Consequently, both aspects affect the aspect ratio of the recycled fibers [37]. Fibers from tires are contaminated with high percentages of rubber. They must be cleaned and shortened in homogeneous size distribution. These processes can damage the mechanical properties of the fiber and because of this, some authors have studied the possibility of using them without cleaning or without shortening [51,52,53,54]. The interest of using recycled steel fibers in concrete formulation is increasing due to the lower reinforcing cost compared to industrial steel fibers. Details on the use of waste tires in concrete formulations have been reviewed recently [41], but this paper does not cover hybrid formulations.

The use of RGF in R-FRC as a reinforcing fiber is less common than steel. In this case, it is mainly used as glass powder as a pozzolanic aid to replace part of the cement powder [55]. RGF is obtained mainly from other building composites wastes, for example, waste thermoset composite, insulator materials, or end-of-use glass fiber reinforced polymers. The combination of RGF with polyester fibers extracted from the same thermoset composite and with the waste powder resulting from the recycling process can increase tensile strength up to 80% compared to plain concrete. Another possibility is to combine RGF with CF, obtained from recycled polymer core conductors, in order to reduce the weight of the construction element and to provide hardness to the composite’s surface [56,57].

RPF are recovered from many different sources with municipal waste the most common, especially from high density polyethylene (HDPE) and polyethylene terephthalate (PET). Many studies have demonstrated the possibility of using different types of RPF in R-FRC production, such as PET [58,59,60,61,62,63,64], PP [65], polyvinyl chloride (PVC) [66], high/low density polyethylene (HDPE/LDPE) [59,67,68,69], polyamide (PA) [70], thermosetting plastic [71], shredded and recycled plastic waste [72,73,74], waste carpets [75,76] and expanded polystyrene foam (EPS) [77], among others. Recycled plastics can be added as aggregates, fillers or fibers in FRC, each having different functions. Gu and Ozbakkaloglu (2016) [43] have published a deep review on this topic. Their main conclusions were: (i) the use of recycled plastics in the form of aggregates decreased FRC density while in the form of fibers had a not significant effect on density; (ii) the polymeric fibers improved mechanical properties (flexural, compressive and tensile strengths) if their volume percentage was lower than 1% and decreased shrinkage; (iii) PP was the most efficient reinforcing polymer; and (iv) the roughness and irregular shape of fibers increased fiber-matrix interaction.

### 4.2. Physical, Mechanical and Chemical Properties of Recycled Fibers

The dimensions and properties of the recycled fibers mainly depend on the original source and the recycling process. For example, mechanical recycling of tires, shredding, cutting the fibers and producing short thin fibers, while pyrolysis of tires keeps the original steel fiber dimension [41]. Table 1 summarizes the properties of common recycled fibers used in hybrid R-FRC.

The RTSF size distribution is broad and they are partially deformed, which increases mechanical agglomeration. Most of the researchers that use RTSF in hybrid R-FRC, do it for structural application. Steel is one of the most promised recycled fibers because of its high strength and ductility, especially in RSF from tires. In this case, the flexural and tensile strengths are higher than many of the industrial steel fibers, because of their different composition [78]. Steel has poor chemical strength, compared to other materials, which is the main drawback of these fibers. However, its durability increases if it is covered by rubber. The chemical strength depends on fiber morphology and decreases with increasing specific surface. Despite of that, some authors have used micro-steel fibers because of their good performance in crack control and in improving post cracking behavior [79,80,81].

Although there are several works on the use of virgin glass fibers in hybrid FRC, especially combined with synthetic polymeric fibers, for example PP, there are not many researchers that have used RGF in hybrid R-FRC formulations. RGF are mainly designed to be thermal and electrical insulators and to be highly compatible with polymeric matrixes. They have the drawback of their poor ductility, which can be compensated by a combination with polymeric fibers. Alkali resistant glass fibers have the advantage of their high chemical strength and their high compatibility with the cement matrix, but the composition of RGF is different from the alkali resistant glass fibers, which affects the R-FRC durability. RGF are mainly recovered from building waste, for example insulator composites and automotive wastes. Furthermore, while the morphology of virgin fibers is homogeneous and they are well dispersed, RGF are heterogeneous, form bundles and they can contain part of the polymer matrix. These facts affect their reinforcing ability [56,57,82].

As it is well known, the morphology and properties of polymeric fibers vary notably with the nature and source of the fibers [44,83]. The most used RPF in R-FRC are PP and PET, due to their high availability in municipal wastes. RPF are often extruded again from chips made from the recovered plastic wastes. Therefore, they can have the same morphology than the virgin fibers. However, the chemical composition differs due to both the presence of impurities and to the shortening of polymeric chains, which reduces the molecular weight. This decreases their mechanical properties [84].

### 4.3. Effect on Fiber-Cement Properties

The effect of recycled fibers on FRC properties depends on their morphology, chemical composition and mechanical properties, which are determined by the waste source and the recycling process. Apart from their mechanical properties, the reinforcing efficiency of fibers is determined by their homogeneous dispersion and the bonding ability with the matrix. Therefore, the selection and properties of waste fibers is key to assure an optimal FRC reinforcement performance and therefore to reach the same performance of the standard industrial fibers reinforced composite [97].

**Figure 2:**
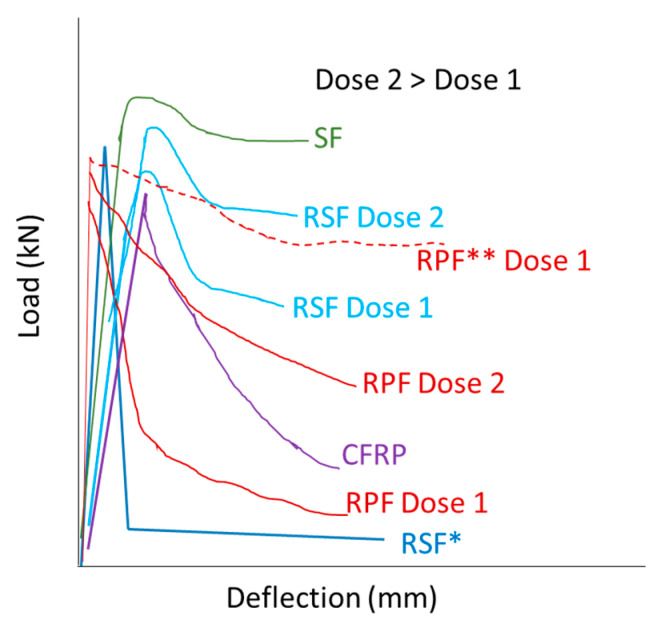
Stress–strain behavior of FRC during bending test. Effect of the dose, nature and origin of the reinforcing fibers. * RSF from machining process discards. ** tension zone confined RPF.

The kind, origin and dose of fibers affects the stress–strain characteristics. Figure 2 shows a general load–deflection scheme based on the bending test load–deflection curves obtained by different authors for FRC [98,99,100,101]. Load is proportional to deflection for low deflection values, indicating elastic behavior. After reaching a maximum load (load capacity) the material can failure or absorb some energy keeping part of the strength after the first crack. In the case of industrial steel fibers (SF) and RSF the material bears some of the maximum loads as shown by a constant load value with increasing deflection, which shows some plastic behavior. In general, a high percentage in fiber volume leads to an increase of the load capacity up to a maximum value, but it does not affect elastic modulus. In case of RSF, the residual load after cracking also increases with reinforcing fibers dose above all to an increase of the residual strength and toughness. In fact, similar behavior was observed for R-FRC reinforced with a similar dose of RSF (from tires) and industrial steel fibers [102], confirming the predominant effect of volume fraction compared to nature of fiber in that case. However, a different stress–strain behavior was obtained when RSF came from machining process discards [98]. These RSF increased brittleness and stiffness of the FRC, causing a detrimental effect on mechanical properties. This is mainly due to the mechanical weakening of steel fibers during the machining process and their pulling out during bending test [98].

The reinforcing effect of RPF is often lower than that for RSF from tires, but they are also able to increase load capacity and residual strength, although a minimum dose could be required to observe those effects. For example, PET fibers only increase load capacity, toughness and energy absorption of plain concrete at doses of 0.18% and higher [99]. Similar observations were reported for other polymers. Ogi 2005 [101] observed that load capacity and residual strength of FRC increased with dose of recycled crushed carbon fiber reinforced plastic. However, Anandan 2021 [103] tried recycled PET (bottles) volume fractions lower than 0.15% and observed that while the unreinforced concrete failed at first crack (it broke) the FRC had a residual strength after the first crack that increased with fiber volume fraction even when it was as low as 0.05%. Confining the RPF in the tension zone of the RFC had a similar or even higher effect than increasing RPF volume fraction.

In the case of steel fibers, there are controversial results when comparing published works. Recycling affects their morphology, but it does not necessarily cause a detrimental effect on the mechanical properties of the fibers [104,105]. For example, industrial hock-end fibers are very efficient in improving tensile strength, because they are specifically manufactured with that purpose, to prevent pull-out. However, recycled fibers are not specifically designed for this kind of reinforcement, which affects the breakage mechanism in splitting and bending failures when fibers were recovered from machining process discards. This causes the energy absorbing efficiency after cracking to be about six times lower [98]. The heterogeneous fiber dimensions and geometry also contribute to the detrimental effect on concrete consolidation. To solve this, Grzymski et al. (2019) [98] proposed to recycle steel fibers through the metallurgical industry as a more convenient approach. On the contrary, several authors have found significant improvements in tensile and flexural strength when recycled steel fibers were used for reinforcing plain concrete, reaching similar reinforcing efficiency that non-hook-end industrial fibers [81,106]. Furthermore, the post-cracking behavior is notably affected by using recycled fibers with or without combining them with industrial ones [37,107,108,109,110]. One of the key factors to get better performance than industrial ones, is the aspect ratio of fibers. Alarger aspect ratio is linked to better crack control and higher cracking toughness [37,104].

When glass fibers are used as reinforcement in FRC, chemical composition is a key issue. Glass fibers in cement matrix can produce an expansive gel due to the highly alkali environment, in long-term affecting the durability of concrete. Due to this, especially alkali resistant glass fibers are preferred as reinforcement in FRC. However, RGF are recovered from composites with other kinds of matrix, for example thermal insulators, reinforced polymers, electrical insulators composites, among others; they do not have such a high durability on matrix cement. However, RGF recovered from woven fiber sheets have been used by Mastali et al. (2016) [111] with high improvements in toughness and flexural strengths compared to plain concrete and with negligible alkali–silicate reaction expansion. RGF from circuit board manufacture have been proved to improve compressive strength and sulphate resistance of concrete [112].

In general terms, the presence of recycled plastic waste (fibers or aggregates) produces a reduction of density (5–25%) of concrete or mortar [59,60,62]. This is useful in applications that require lightweight materials. For example, the addition of waste PET fiber to the plain precast concrete panels allows reducing the thickness of the panels and improving their mechanical properties (i.e., impact resistance and load at rupture) providing an economical production of precast concrete panels [95]. The addition of recycled PET fibers induces a clear improvement of the flexural strength up to 30–40% at age of 28 days by adding 1–1.5% fiber volume [59,60,113,114].

The use of PET fibers decreases notably workability of concrete independently of their geometry and dimension [59,60,115,116]. On the contrary, their use has a significant contribution to the mechanical properties that vary with the geometry and dimensions of the fibers. Flattened-end sheet fibers have a better bonding behavior in the concrete matrix, showing a significant flexural improvement over the straight slit fibers. In addition, recycled PET fibers increase the bending strength about 100%, 30% and 50%, at 7, 28 and 63 days, respectively [60].

However, the incorporation of recycled PET has not a clear effect on compressive strength. Guendouz et al. (2016) [59] found that compressive strength of sand concrete increased up to 25% with 1.5% of plastic fibers content. However, de Oliveira and Castro-Gomes (2011) [60] and Pelisser (2012) [99] did not observe a significant change of this magnitude and other authors [61,117] reported a reduction of 1–9% by adding 0.5–1.0% fiber volume fractions, compared to the non-reinforced specimens. Additionally, Irwam et al. (2013) [118] demonstrated that the addition of PET fibers decreased the compressive strength and splitting tensile of concrete specimens too. The reason for these controversial results could be related, among other variables, to the PET fiber dose [119]. Marthong (2015) [116] found that the improvement in compressive strength also varies with the geometry and dimensions of the fibers. Flattened-end slit sheet fibers showed a significant improvement over the straight slit sheet fibers in terms of compression strength, load carrying capacity and energy dissipating capability [120]. Moreover, smaller fiber dimensions exhibited a higher compressive strength and better energy dissipation capacity compared to larger fibers [116].

Fadhil and Yaseen (2015) [95] found that the addition of 1.0% of recycled PET fibers from plastic beverage bottles increased impact resistance and failure strength of precast concrete panels by 157% and 34%, respectively, compared with plain panels. In addition, the failure in PET fiber reinforced concrete panels is fiber pull-out and the panels remain together in one broken piece, whilst plain concrete panels exhibit total disintegration and shattering.

Guendouz et al. (2016) [59] studied the use of recycled LDPE powder aggregates, with a 2 mm maximum size, obtained by compressing and crushing of old jerry cans in sand concrete manufacturing. They found that recycled LDPE powder, as a partial replacement of sand, contributed to an increase in the compressive and flexural strength of about 30% with an addition of 20% of plastic powder content (in volume fraction). However, other researchers have reported that the addition of recycled plastic aggregate, as a partial substitution of natural aggregate (sand) in cement-based composites, can affect negatively the mechanical properties [58,62,121] due to the poor bond between the plastic aggregates and the cement matrix. However, the use of LDPE fibers improves mechanical properties such as flexural strength and plastic shrinkage [122].

### 4.4. Effect on Fiber-Cement Production Process, Economy and Sustainability

The most common effect of using fibers is the reduction of workability of fresh concrete regardless the nature of the fibers [1,41,123,124]. This has been explained by the irregular morphology and high specific surface of the fibers [100], which increases water demand. Decreasing workability complicates the homogeneous fiber dispersion within the cement matrix, which could justify the reinforcement differences observed. For each nature, the morphology and flexibility of fibers are key factors in workability, which decreases with an increasing aspect ratio. The replacement of virgin fibers by recycled ones will affect the workability if their aspect ratio, shape or dose are different form the virgin ones.

Furthermore, many of the recycled fibers tend to mechanically knit during mixing in fresh concrete, which hinders their dispersion in the matrix and reduces workability. This phenomenon is called “balling” and it is favored by broad size distributions and a variety of shapes [40,95]. To avoid balling, fibers must be gradually added during fresh concrete paste mixing.

To solve the workability limitations and the fiber balling, while keeping a high compressive strength, Chu et al. (2021) [125] proposed modifying the FRC production process, by means of forming a skeleton structure with fibers and coarse aggregates that were filled by flowing fresh cement. This kind of product, called “infilled cementitious composite”, allows a significant increase of fibers and coarse aggregates, notably reducing the required fresh cement. Consequently, the carbon footprint would decrease.

Guendouz et al. (2016) [59] and de Oliveira and Castro-Gomes (2011) [60] studied the use of recycled PET fibers, with 35–40 mm of length and 0.5 mm of thickness, obtained by mechanical cutting of PET bottles, as fiber-reinforced sand concrete. The workability decreased about 60% with the addition of recycled PET fibers; however, it increased with recycled powder plastic aggregates (for example, ~40% of slump increment with 30% of recycled LDPE powder) [59]. Similar results have also been reported by Irwam et al. (2013) [118] and Fadhil and Yaseen (2015) [95], confirming a significant reduction in the workability of the concrete by the introduction of recycled PET fibers, due to the reduction of free water, the increment of the stability and a better cohesion in the mixes containing fibers compared to the plain concrete. Workability decreased, when the percentage of the waste PET fiber increased, due to balling [94]. Additionally, Marthong (2015) [115] and Marthong and Sarma (2016) [116] have demonstrated that PET fiber geometry had a small effect on the workability of concrete, although the use of smaller dimensions of fiber slightly improved this parameter.

Therefore, it is clear that the concrete composition must be optimized considering the recycled fibers characteristics to achieve the required workability and a good fiber dispersion [96]. To improve homogenization, ordinary mixers maybe replaced by planetary mixers [102]. Other complementary approaches are to increase the superplasticizer dose [126], to keep constant the fresh cement workability, or to increase the w/c ratio, which affects the mechanical properties of the concrete.

Air content can increase with fibers addition too, especially if the specific surface is high and the fiber is grouped in bundles with aspect of fluff [127]. This morphology is more likely in the case of recycled fibers than in the case of virgin ones. An approach to minimize air entrapment is to soak the fibers before their addition. This contributes to reducing the effect of the fiber on workability too. Furthermore, the morphology of coarse aggregates can also contribute to air entrapment as observed by Zotov et al. (2018) [128], who analyzed the steel fibers and air voids distributions in SCC.

Some authors have observed a beneficial effect of the fiber’s presence on segregation prevention. Fibers contribute to block segregation of particles in cement, providing they are well dispersed [41,124]. One of the most important effect of using recycled fibers in the FRC process is the reduction of cost. Reinforcing fibers is one of the most expensive components of FRC, especially for steel fibers. Onuaguluchi et al. (2018) [81] studied the economic feasibility of replacing virgin hock-end steel fibers by scrap tire steel fibers in FRC formulation by means of a cost–benefit analysis. They concluded that complete replacement of hocked-end fibers by recycled fibers was beneficial for FRC that contain 0.35% of fibers if the cost of virgin fibers is 5 times higher than that of recycled fibers.

Nevertheless, the main benefit of using recycled fibers is the reduction of the environmental impact. Several authors have studied the environmental sustainability of the R-FRC process including Life Cycle Assessment (LCA) [91,129,130]. Yin et al. (2016) [91] studied the LCA of the R-FRC comparing virgin, domestic and industrial recycling PP. They concluded that, although both recycled PP fibers (domestic and industrial) had a lower environmental impact than the virgin did, the use of industrial recycled PP highly reduced the carbon print, water print, energy consumption and eutrophication impact compared to the domestic ones. This is because manufacturing recycled PP fibers from domestic waste is a more complex and energy intensive process, requiring waste collection and sorting, cleaning, reprocessing and fiber production.

## 5. Hybrid R-FRC

This section focusses on hybridization with homogeneous nature fibers, i.e., metallic-metallic, polymeric–polymeric fibers. Therefore, all the combined fibers have a similar affinity for the matrix and their differences are mainly based on the fiber source (recycling process or industrial manufacturing), the kind of polymer or the fiber morphology. The effect of hybridization with heterogeneous fibers, with a different affinity for the matrix, is more complex and it will be reviewed in Section 6.

### 5.1. Metallic Fibers

Several studies have evaluated the combination of industrial and RMF, mainly obtained from waste tires, in the performance of hybrid R-FRC (Table 2).

Caggiano et al. (2017) [37] have reported that small amounts of mixed-fibers (industrial and RMF) (0.75–1.00% volume fraction of total mixed-fibers content) slightly increased the compressive strength by 5–10%, whereas this effect disappeared when the number of fibers was higher than a certain level (i.e., 1.25% in volume). This detrimental effect on compressive strength due to the high number of fibers had been previously reported [131,132]. Moreover, at lower fiber content (i.e., 0.5% volume fraction), the compressive strength of hybrid R-FRC was mainly controlled by the matrix properties and, therefore, no significant difference was observed in terms of compressive strength between the R-FRC and the plain mixture [106]. The detrimental effect on compressive strength has been explained as a function of the entrapped air [38,85], which increased with the volume fraction of fibers. Baricevic et al. (2017) [38] used the recycled steel fibers from tires without cutting them to avoid damage of their mechanical properties. They observed that there was a correlation between the entrapped air and the effect on compressive strength. High improvements on compressive, tensile and flexural strengths were observed for self-compacting concrete and high w/c ratio (0.76). In this case, the total volume fraction was 1.5%, and the best results for hybrid R-FRC was obtained when recycled steel fraction was 0.5%. The anchoring effect of the hooked ends of industrial steel fibers contributed to explaining these good improvements [107,108].

It is well known that the flexural toughness of the FRC can be evaluated by determining both first crack strength and post-cracking flexural behavior (in terms of equivalent post-cracking strengths and/or ductility indices) to evaluate the flexural behavior until the first cracks appear and after cracking, respectively. Some studies have reported that the presence of recycled fibers in the hybrid R-FRC has not had an important influence on the first crack strength [132,133,134] but in other studies a clear increment of this parameter has been reported [37,106]. Caggiano et al. (2017) [37] demonstrated that the combination of recycled and industrial metallic fibers increased the first crack strength by 20.5% in comparison with a mono-fiber composite containing an equal number of total fibers (0.75%) but with only industrial fibers [37]. Moreover, the highest first crack strength value (4.22 MPa) was obtained when higher number of mixed fibers (1.25%) in a proportion 70:30 (recycled fibers: industrial fibers) was added to the mixture, achieving an increase of 30% in comparison with the reference mixture containing 0.75% of industrial metallic fibers [37]. Martinelli et al. (2015) [106] reported that the first crack strength of hybrid metallic R-FRC with 0.5% (in volume) prepared by replacing 50% in weight of industrial metallic fibers for recycled ones, increased by 10.4% in comparison with an FRC with only industrial metallic fibers.

On the contrary, a significant decay in the post-cracking flexural behavior has been reported by several studies when virgin steel fibers are partial or total replaced by RTSF [37,106,133,134]. Martinelli et al. (2015) [106] found a substantial reduction in the equivalent post-crack resistances, defined in the standard UNI-11039-2 [137], around 20% as a result of 50% replacement of industrial fibers with recycled ones [106]. Moreover, the mixtures reinforced with only RTSF could reduce the post-crack resistance strength by more than 50%, compared with the ones with the same number of unrecycled fibers [70]. Similar results were found by Bjegovic et al. (2013) [133], who concluded that the hybrid composite cost savings could reach up to 50% with respect to industrial steel FRC and the mechanical properties would be similar if 5% of recycled rubber was added to improve ductility and post-crack behavior of the R-FRC.

In general terms, the higher the fraction of recycled fibers, the more significant the reduction in the post-cracking toughness (in terms of equivalent post-cracking strengths and/or ductility indices) observed in the four-point bending tests [106]. The main reasons that explain this reduction in the bending behavior are the usually lower aspect ratio, non-straight and non-hooks of RTSF compared to the virgin metallic ones. Caggiano et al. (2017) [37] have also found that the ductility was significantly influenced by the fiber contribution and fraction. They observed that higher total amount of fibers in the hybrid R-FRC slightly increased ductility [37].

The post-cracking response of all composites (mono-fiber and hybrid metallic fibers composites) for small crack openings (those related to D_0_ values) were defined as a plastic type. However, the presence of RMF in the composites turned the post-cracking behavior at ultimate state from hardening (when only industrial fibers were used) to a plastic type [37]. This post-cracking response of hybrid metallic R-FRC, almost comparable with the one obtained for mixtures with only industrial ones, was due to the high bridging ability of RMF, with a larger aspect ratio (~110) than that of the industrial ones (~60). Therefore, the performance of RMF is highly linked to the aspect ratio; this is also observed in the previous results reported by Martinelli et al. (2015) [106].

Another way to improve post cracking behavior is the use of steel cords in R-FRC. Hu et al. (2018b) [136] observed that the combination of recycled steel cords with recycled steel short fibers allowed obtaining specimens with deflection hardening behavior, which makes them useful in those structural applications where a bending risk exists [138]. Furthermore, it significantly enhanced ductility and flexural toughness compared to plain concrete with an increase in post-cracking strength up to 103% compared to the FRC with manufactured short fibers.

Although steel fibers are the most used in the formulation of hybrid metallic fibers R-FRC, there are some studies combining different metals too. For example, Naser et al. (2020) [90] combined short copper fibers, recycled from wastes of electrical connection wires, with long steel fibers recycled from galvanized binding wires. They tried different percentages and combinations of fibers and the highest flexural and tensile strengths (10.7 MPa and 5.94 MPa, respectively) were obtained by combining 0.45% copper fibers with 0.105% steel fibers. On the other hand, the use of 1.5% copper fibers caused the highest increases in compressive strength, reaching 71 MPa, and flexural strength was limited to 9.3 MPa. However, the use of 1% copper fibers allows reaching compressive, flexural and tensile strengths of 69.3, 8.6 and 5.3 MPa, respectively, with a 33% lower fiber cost. Moreover, the use of 0.3% copper fiber with 0.7% steel fibers gave a similar compressive strength (67.2 MPa) with lower cost and much higher flexural strength (10.1 MPa) and the same tensile strength (5.3 MPa). Therefore, in this case, hybridization not only saved costs, but it improved flexural strength.

### 5.2. Synthetic Polymeric Fibers

Limited studies have been reported on the use of recycled low costs fibers, mainly PET and PP fibers, to partially replace non-recycled fibers in cement composites (Table 3; Table 4). Cheng et al. (2017) [94] studied the replacement of coarse aggregate by walnut shell as an eco-friendly strategy to develop a lightweight shotcrete. The low specific gravity of crushed walnut shell (1.02 g/cm^3^) was much lower than that of crushed gravel (2.64 g/cm^3^). However, the replacement of 25%, 50% and 75% of gravel by walnut shell reduced the compression strength by 28.7%, 41.9% and 63.6%, as well as the splitting tensile strength by 28%, 47.93% and 68.8%, respectively. Therefore, Cheng et al. (2017) [94] evaluated the addition of recycled and non-recycled fibers to counteract the loss of the mechanical properties of the shotcrete containing walnut shell while reducing the cost of the product and solving waste disposal problem, in case of using recycled fibers. Thus, non-recycled PP (nPP) fibers composite and hybrid composite, with nPP and recycled PET (rPET) fibers, were evaluated in terms of mechanical properties (splitting tensile strength and compressive strength), pumpability and shootability (rebound rate and build-up thickness). Results show that single fiber addition (nPP or rPET) increased the splitting strength of the mixture (with 35% aggregate replacement of walnut shell) by 27%. Moreover, the dual combination of rPET + nPP (0.045 + 0.325% w/v) fibers produced higher splitting strength than single fiber addition (56% of splitting strength increment compared to the plain mixture without fibers). Compressive strength slightly decreased with a single fiber addition, but it increased when rPET+nPP fibers were added due to a mixture because the presence of fibers with different dimensions has a positive effect on mechanical reinforcement [3,116,118]. Besides mechanical properties, the addition of fibers reduced slump by 18.6%, 8.6% and 16% for nPP, rPET and rPET+nPP mixtures, respectively. As a consequence of this slump reduction, pressure drop increased by around 20%, which is not beneficial for flowing fresh concrete in pipes. However, the addition of fibers improved the shootability, in terms of reducing rebound rate and increasing build-up thickness, achieving the highest build-up thickness (130 mm) with the hybrid composite design [94].

Combining fibers with different sizes allows to increase the reinforcing effect too [15], but it could have a strong effect on workability due to its dependence on fiber morphology. The effect of different sizes of recycled fibers on workability and mechanical properties of concrete has been evaluated by Ogi et al. (2005) [101]. They studied three different sizes (large, medium and small) of recycled and crushed carbon fibers reinforced plastic (CFRP) pieces in two size testing specimens (large- and small-sized specimen). They found that slump value largely decreased with increasing CFRP content. However, this reduction in workability could be easily overcome by adding a superplasticizer (like polycarboxylic acid), which improves fluidity of the mixture. Results show that the size ratio of CFRP pieces to specimen for compressive and flexural tests was a key factor for concrete reinforcement. In general terms, the strength of large-sized specimens or specimens reinforced with small or medium CFRP pieces increased with increasing CFRP content due to the “size effect”, thereby strength tended to depend on the local distribution of CFRP pieces. For that reason, small-sized specimens with large and medium CFRP pieces as a reinforcement agent could have lower strength values than the plain concrete without fibers.

According to Schmidt and Cieślak (2008) [139], the durability of hybrid R-FRC could be predicted by using the method of assessing surface properties (contact angle and free surface energy) of the different recycled fibers added to the mixture. They found that recycled polyamide fibers (rPA) were more strongly bonded to the concrete compared to recycled PP fibers (rPP) but, on the contrary, rPP were more water-resistant. Therefore, the presence of both recycled fibers (rPA and rPP) has a synergistic effect forming strong and water-resistant bonds with concrete.

For enhancing the alkali resistance of the rPET fibers and improving the fiber–matrix interfacial chemical and frictional bond, rPET fiber surface could be treated with NaOH solution and a silane coupling agent [141]. Recently, Yu et al. (2018) [141] have also evaluated the performance of strain-hardening cementitious composites (SHCC) when 2.0% of PVA fibers (in volume fraction) were partially or totally replaced by treated rPET fibers (T-rPET) or untreated rPET fibers (U-rPET). As previously reported by Choi et al. (2012) [140], compressive strength was not affected by partial replacement of PVA with rPET when hybrid fiber composites were cured at 28 days, reaching values around 35 MPa. However, after accelerated aging, hybrid fiber composites (PVA + rPET) showed higher compressive strength (54–63 MPa) than composites with only one type of fiber (PVA or U-rPET or T-rPET) in their structure (49–50 MPa). Moreover, slightly higher compressive strength was observed in hybrid fiber composites with U-rPET fibers compared to T-rPET fibers. Yu et al. (2018) [141] also observed a reduction in both tensile strength and ultimate tensile strain when more PVA fibers were replaced by rPET fibers. The SHCC with only PVA fibers (2.0%) showed the highest tensile strength (5.17 MPa, for 28 day standard curing and 6.15 MPa, after accelerated curing). However, when 25% of the PVA fibers were replaced by rPET, the tensile strength was 4.44 and 4.35 MPa for U-rPET and T-rPET, respectively, after 28 day standard curing, and 4.6 and 4.53 MPa, respectively, after accelerated aging. Although Choi et al. (2012) [140] have studied a similar PET replacement level (20%), the tensile strength and the ultimate tensile strain reported by Yu et al. (2018) [141] were almost twice as high. Moreover, the utilization of rPET fibers in SHCC significantly reduced the environmental impact and material cost by decreasing of around 20% in embodied energy and about 40% in material cost when half of PVA fibers were replaced [141].

Some researchers have evaluated the use of rPP fibers and recycled coarse aggregate as replacement agents of non-recycled fibers and natural coarse aggregate, respectively, in the production of reinforced concrete beams [142,143]. In general terms, in absence of fibers and the addition of a recycled coarse aggregate reduced the compressive strength, the splitting tensile strength and the elastic modulus by around 19%, 7% and 19%, respectively, when 30% of the natural coarse aggregate was replaced [142,143]. However, the presence of a mixed-fiber admixture, composed of rPP and acrylic fibers, increased the mechanical properties, cracking load and yield load of recycled concrete beam to different extent achieving the highest reinforcing effect when the hybrid fiber content was around 0.15–0.20%. Moreover, the mid-span deflection of concrete beams decreased with the increase of hybrid fiber content, which indicates that hybrid fibers contribute positively to the crack resistance [142]. Recently, Cui et al. (2019) [143] have demonstrated that a replacement of 25% of PA fibers by rPP fibers slightly improved the compressive strength and the elastic modulus by 1.1% and 5.3%, respectively, of concrete beams (in which 30% of natural coarse aggregate was replaced by recycled coarse aggregate) in comparison with a single fiber addition with only PA. Moreover, even though 75% of PA fibers were replaced by rPP, the splitting tensile strength was quite similar to the one obtained by only using PA fibers (3.79 MPa). Furthermore, they found that single-mixed fiber (PA or rPP) could effectively improve the crack resistance of concrete beams but the combination of both fibers was not beneficial for the crack resistance. The most unfavorable scenario was when PA and rPP fibers were mixed in an equal quantity due to the large amount and non-uniformity of the mixed fiber, resulting in a heterogeneous distribution and clustering of the fibers that produce more defects in the concrete [143].

SHCC is a type of engineered FRC with high tensile strength, ductility and enhanced properties due to the fiber-bridging action of fine multiple cracks as a consequence of the even distribution of the fibers. Therefore, they present a tensile strain hardening after the first cracks occur. Some researchers have studied the addition of recycled fibers to partially or even totally replace non-recycled fibers in SHCC to reduce the cost and the negative environmental impact associated with the use of industrial fibers instead of non-recycled materials. For example, Choi et al. (2012) [140] have studied the addition of alternative recycled materials such as recycled sand, fly ash (FA) and rPET fibers to partially replace silica sand, cement and PVA fibers, respectively. Results showed that compressive strength was not affected by partial replacement of PVA with rPET (23.48 MPa using 2 wt.% PVA vs. 24.60 MPa using 1.6 wt.% PVA + 0.4 wt.% rPET). However, tensile strength and flexural strength decreased by 39.7% and 10.4%, respectively, due to the inferior mechanical characteristics of rPET fibers compared to the PVA fibers (tensile strength = 953 and 1600 MPa; elastic modulus = 11 and 40 GPa, for rPET and PVA, respectively). Considering the other recycled materials, FA reduced the compressive strength of SHCC, but improved the flexural and tensile strengths due to an improvement in chemical bonding strength at the interface between the fibers and cement matrix. In addition, recycled sand increased compressive strength due to its larger grain size compared to that of silica sand; however, higher replacement of 50% reduces the flexural strength of SHCC.

## 6. Complex Hybrid R-FRC

The use of fibers with different natures allows getting an advantage from the interesting properties of both types of fibers while minimizing the cost. The different nature of fibers implies different affinities with the matrix and therefore, a more complex integral behavior. Most of the studies about complex hybrid R-FRC involve steel fibers and synthetic polymeric fibers, and in the majority both kinds of fibers are recycled from waste (Table 5; Table 6). Recycled fibers usually have lower reinforcing potential than virgin ones, as for example recycled polymers, requiring virgin fibers or other different nature fibers, recycled or not, to complete reinforcing effects while providing other interesting properties to the composite, as for example ductility, chemical strength or dimensional stability. Furthermore, the use of different types and size of fibers is a successful strategy to control cracks at different sizes and during different stages of curing. The complex hybrid R-FRC are classified in three types, according to the nature of reinforcing fibers: (i) metallic fibers—synthetic polymeric fibers R-FRC, which are the majority; (ii) metallic fibers—glass fibers R-FRC; and (iii) carbon fibers—glass fibers R-FRC.

One of the first studies on complex hybrid R-FRC was carried out by Meddah and Bencheikh (2009) [97]. They observed that the use of waste steel fibers decreased flexural strength (6.4 MPa) while it did not improve compressive strength, whereas PP fibers increased flexural strength but reduced compressive strength. The hybrid FRC had a higher flexural strength (9.5 MPa) than that for any of the single fiber FRC, and similar compressive strength than the FRC with waste PP fibers (26 MPa); but compressive strength decreased 25% (24 MPa) compared to unreinforced mortar (30 MPa). The best performance was achieved by the combination of short (3 mm) and long (6 mm) waste metallic fibers. The main benefit was, in this case, the increase in flexural strength and the decrease of cost and environmental impact. Yin et al. (2016) [91] analyzed the LCA of hybrid concrete footpaths with a steel mesh and reinforcing PP fibers. They studied three alternatives: virgin PP fibers and recycled PP fibers from domestic and industrial waste, and they concluded that the use of recycled PP from industrial waste allowed obtaining the highest environmental benefits contributing with a 50% reduction of CO_2_ emissions, 29% water savings and 78% oil equivalent natural resources saving, with respect to the alternative of using virgin PP fibers.

Mastali et al. (2018) [108] also carried out a cost analysis and optimized the hybrid reinforcing for self-compacting concrete considering the effect on mechanical properties, the environmental impact and the cost of the composite. They used recycled steel fibers and virgin PP fibers and compared it with single reinforcement and with the use of industrial steel fibers, considering all the combinations. They observed that the best properties were obtained with industrial steel fibers and with the hybrid industrial and recycled steel fibers. However, the hybrid composite with PP and recycled steel fibers had similar mechanical properties than that with recycled steel, but the use of PP fibers reduced composite cost and composite damage under fire because PP melting made the pressure release easier [82]. Similar hybrid fiber reinforcement was used by Mastali and Dalvand (2017) [144] but with higher mechanical performance. In this case, they did not use coarse aggregates in SCC and w/c ratio was lower. Coarse aggregates affect the fibers distribution in the matrix and the volume and distribution of air entrapped in the SCC. Zotov et al. (2018) [128] observed that irregular aggregates might reduce the orientation of fiber, induce heterogeneous voids distribution and increase stress concentrations near their edges. This could reduce fiber reinforcement performance when coarse aggregates were used in SCC [144]

Karthik et al. (2015) [145] observed that in the case of structural applications, the steel fibers worked as the main reinforcing material while the polymeric fibers mainly preserved the material after crack starting. They concluded that the performance of recycled PET was lower than that for virgin PP fibers in the complex hybrid composite due to the lower interaction of steel fibers with PET fibers. Therefore, many other researchers have tried the combination of steel fibers with PP fibers with at least one of them obtained from waste.

**Figure 3:**
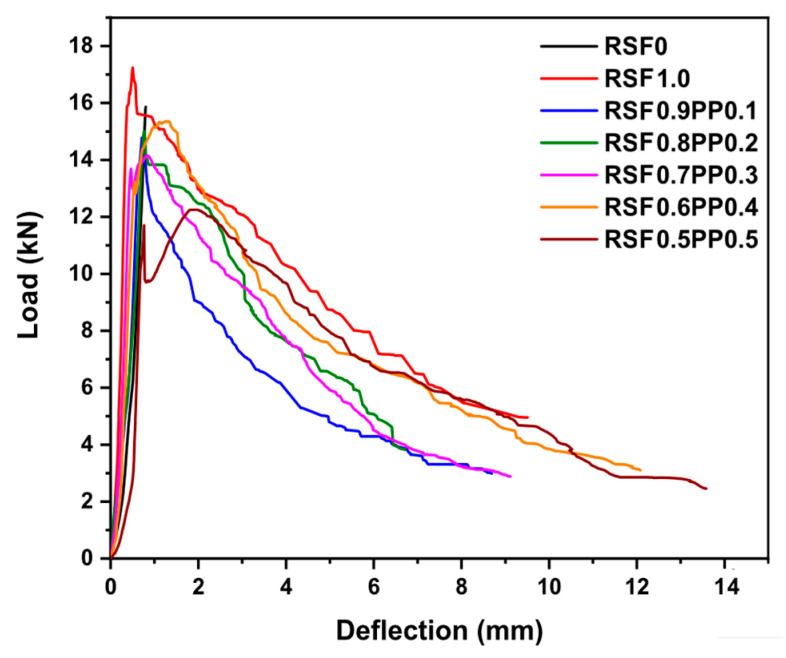
Load deflection curve for complex hybrid FRC. Adapted from [89].

Waste steel fibers decrease workability of the fresh paste and it affects the product properties or the consumption of superplasticizers. As already mentioned, this effect is related with the non-uniformity of the recycled steel fibers and with the aspect ratio. One of the benefits of hybridization with PP fibers is the improvement of workability by replacing part of the waste steel fibers. This is one of the results obtained by Zhong et al. (2020) [89] who observed that workability improved by 38.9% to 66.7% when part of the waste steel fibers was replaced by PP fibers. It resulted in a good approach, for a total fiber volume fraction of 1%, providing that the volume fraction of PP fibers was under 0.3%. The other way, the compressive, splitting tensile and flexural strengths decreased due to weak bonding between PP and cement. This was also observed by Mastali and Dalvand (2017) [144] for the same nature of fibers in self consolidated concrete. Despite of this, all the hybrid FRC containing RSF and PP fibers had a high residual strength after first cracking (Figure 3). This, and the load capacity, increased with volume fraction of PP fibers, reaching similar values than FRC reinforced with RSF [89].

Waste tires are one of the most studied sources of recycled fibers for hybrid R-FRC because they contain metallic and polymeric fibers in one source and the rubber can be used to replace part of ground aggregates in concrete with the advantage of its lower thermal conductivity. Serdar et al. (2014) [150] studied the possibility of using each one of those recovered materials in cement composites products. They concluded that rubber reduced the erosion effect of freezing–melting cycles and water adsorption; steel fibers increased ductility of concrete and strength against corrosion; and, finally, textile fibers decreased shrinkage during the hardening process. The combination of the three components could provide interesting properties to the concrete. One of the main drawbacks is the high cost of completely separating the fibers from the tires. Fibers are embedded in rubber and recovering clean fibers requires high energy consumption. Due to that, some researchers have studied the use of the rubber covered fibers in concrete formulations for non-structural FRC with the aim of saving energy and tire treatment cost. Papakonstantinou and Tobolski (2006) [151] observed that the use of these metal fibers covered by rubber increased FRC ductility up to 20% over that without fibers. The synergy effect between hybrid fibers and rubber has been studied too [51,52,133]. Since the interaction between rubber and cement is poor, negatively affecting the mechanical properties, the rubber must be pretreated with alkali before using, for example, by soaking in a Ca(OH)_2_ saturated suspension or by coating with limestone paste [152]. Rubber decreases thermal conductivity, which is a key property in building thermal insulation, but it decreases mechanical properties too. This limits the percentage of rubber used as aggregate in rubberized cements. However, the use of complex fibers covered by rubber makes it possible to increase thermal insulation with a minimum reduction of mechanical properties and with a higher impact energy absorption, because the steel fibers have better interaction with cement [51,52]. Therefore, the use of partially covered complex fibers is an alternative to rubber aggregates in rubberized cement, especially for total volume percentages under 7.7%.

Waste steel fibers can be also obtained from lathes [50,97]. Combining these scrap fibers with waste nylon fibers has been proven to be an efficient reinforcement strategy for high compressive strength concrete (60 MP). The reinforcing effect increased with fiber volume fraction, reaching a maximum performance for compressive strength with a total fiber volume fraction of 1.5%, except in the case of combining steel and nylon fibers with a volume steel/nylon ratio of 2/1. In this case, it reached compressive strength equal or even slightly higher than that for concrete with 1.5% steel fibers but increased both tensile strength and modulus of rupture by 13% and 30%, respectively, over those for steel FRC and 54% and 40%, over plain concrete. However, the highest energy absorption and the best behavior under shear and impact was observed for the composite with a steel/nylon ratio of 1, which indicates that the hybridization ratio must be optimized for each concrete application [148,149]. In these cases, the fresh concrete workability was kept constant by adjusting superplasticizer dose avoiding the effect of poor workability on the composite properties. This could be the key for the good results obtained from this research.

Different results were obtained for hybrid composite with lathe waste steel and recycled PP fibers from bags. In this case, the compression strength of the composite was up to 20% lower than that for plain concrete (30 MPa), but flexural strength increased up to 12% [97]. This is related to the poorer properties of the PP, as shown by the fact that the use of waste steel fibers did not affect to the compressive strength at that volume fraction (1%), and the fact that using industrial PP on waste steel reinforced high-strength concretes (60 and 80 MPa) but had a lower effect on compressive strength [146]. The composition of concrete could have some influence on PP reinforced performance since the effect of PP on the highest strength concrete was the double than that for the concrete with a compressive strength of 60 MPa [146].

Ozerkan et al. (2016) [79] studied the effect of replacing the half of steel fibers with recycled HDPE fibers in self-consolidating concrete (SCC) reinforced with micro-steel fiber. The aim was to reduce microcracks and save costs, while improving municipal waste management. Both fibers led to a similar reduction on compressive strength, around 30%, but no significant effect on compressive strength was observed after curing for 7 days. The effect on flexural strength was only 10%. The main benefits of hybrid fiber reinforcing, apart from the cost savings, was the strong reduction on drying shrinkage and the better interaction of HDPE with the matrix compared to that for steel fibers. Workability of the fresh composite was reduced with hybrid fibers, but that is good for SCC to keep its shape during the consolidation stage.

Some studies combined industrial or recycled metallic fibers with glass fibers for structural applications. Glass fibers have a better affinity for cement matrix than polymers, but they are rigid. The effect of steel–glass hybrid reinforcement on compressive strength of self-compacting concrete is not significant [82]. However, combining polymer and glass fibers notably increased the tensile strength [56] and even more if recycled glass grinding powder was added to the mixture. In this case, the addition of powder to the hybrid FRC mixture increased the compressive strength up to 20%, when a high-speed mixer was used to increase the homogeneity of fiber and powder dispersion in the matrix.

The use of complex hybrid carbon and glass reinforcement made no sense 10 years ago. However, the increasing use of polymer core composite conductors formed by glass and carbon hybrid fiber made the researchers aware about the recycling difficulties of that waste, which is costly and complex because the harsh methods required to separate both materials can severely degrade the mechanical properties of the fibers. The increase of wastes from the use of this kind of composite materials requires developing new uses and Clark et al. (2020) [57] proved the feasibility of using them in R-FRC formulations. They used very small fibers (powder) obtained from milling the polymer core composite conductor. They observed that compressive strength decreased compared to plain concrete, but in less extension than the decrease with virgin fibers due to the small size of the powder that allows better dispersion in fresh cement. The percentage of powder and fibers was one of the highest ones (6 wt.%) compared with other authors that work with less than 2% in volume. This percentage should be optimized to study any possible reinforcing effects.

## 7. Potential Applications of Recycled Hybrid Composites in Civil Engineering

**Figure 4:**
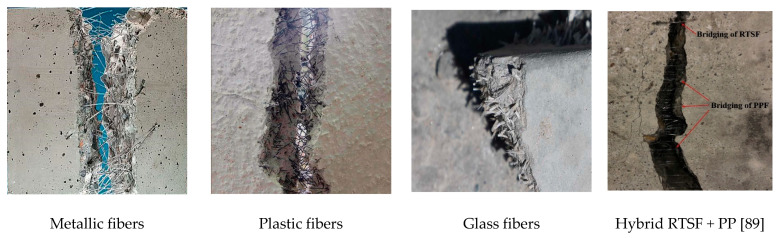
Cement based elements reinforced with recycled fibers used in R-FRC applications.

The results compiled in this review have demonstrated that the use of different recycled fibers from waste (Figure 4) would allow for obtainment of R-FRC with the properties required to be used in different branches within the civil engineering, as it has been suggested by different authors:

- Structural Engineering, for repairing and reinforcement buildings and infrastructures [37,38,80,82,94,101,106,108,142,143,145];
- Transportation Engineering, in the construction transport infrastructures such as high-speed railways [133,146], in pavement applications [85,136,153], or mine roadways [94];
- Material Engineering, as advanced construction materials [140,141] or as materials for construction of thermal storage units for solar plants [70];
- Construction Engineering, as repair mortar [81], for thermal rehabilitation of buildings [51,52];
- Tunnel Engineering, for construction of lined tunnels [150].

In most of the cases, the content of recycled metallic fibers from waste tires with hooked ends of industrial steel fibers or with industrial PP fibers, increases the impact energy absorption, the chemical resistance in seawater and decreases shrinkage of the R-FRC accomplishing the strict mechanical requirements for structural applications.

On the other hand, hybrid R-FRC with recycled glass fibers or recycled plastics, obtained from recycled bottle or carpets, is a good option for applications with high flexural strength and low shrinkage requirements as pavement constructions or design of advanced construction materials with specific properties, e.g., low thermal conductivity.

## 8. Conclusions

Research on the use of recycled reinforcing fibers for producing more sustainable hybrid fiber cement-based materials is very widespread in the last few years. Although current findings prove the valuable use of recycled fibers in hybrid FRC, the knowledge on the potential enhancement of different properties is fragmented. This critical review compared and grouped the different results, to provide a deep understanding on the use of different recycled fibers. Furthermore, the effects of using different recycled fibers, alone or in different combinations, on the different properties of the final cement products as a function of the dosage are quantified. Thus, the use of reinforcing fibers can be optimized according to different final product requirements. From the analysis of the results compiled in this review, it is concluded:

- The highest effort in using recycled hybrid reinforcement has been carried out in the framework of structural applications, where FRC must accomplish strict and tough mechanical requirements. For this application, the combination of steel fibers from waste tires with hooked ends of industrial steel fibers or with industrial PP fibers is the most efficient reinforcing approach. This combination (i) improves the final product: increases impact energy absorption, increases chemical resistance in seawater, and decreases shrinkage; (ii) saves costs; and (iii) reduces the environmental impact. On the other hand, the effect of hybridization on compressive strength can be detrimental. In most cases, there is only a slight decrease, but if polymeric fibers are present the decrease is up to a 30%. However, when different steel fibers are used the compressive strength may increase with respect to plain concrete. Therefore, each fiber fraction must be optimized for each application. Liew and Akbar (2020) [40] have recently concluded that the effect of using recycled steel fibers on compressive strength is not clear yet, which is further confirmed by the results analyzed at this review.
- For applications with high tensile strength requirements, three good options have been identified: (i) hybrid R-FRC with RGF and unsaturated polyester, both obtained from thermoset composites; (ii) recycled PP fibers combined with acrylic fibers; and (iii) the hybridization of recycled PET with industrial PP fibers. The last can be the best option to manage part of the recovered PET from waste PET bottles in R-FRC, with a notable increase in R-FRC tensile strength compared to plain concrete.
- Besides the potential valuable of recycled hybrid FRC, there are several challenges that must be solved for the implementation and consolidation of these processes, which require further research efforts in several topics:
- Challenges related to the optimal morphology of the recycled fibers, because size and shape distributions are too broad to obtain good results at industrial scale. In most studies, researchers have cut or selected the fibers manually, which is not possible in a large scale FRC production. The broad size and shape distribution of fibers reduces workability in excess and favors their mechanical entanglement, increasing the consumption of superplasticizer and challenging the homogeneous distribution of the fibers in the matrix;
- The way to extend the limits of the reinforcing effect of recycled polymers;
- The optimal dispersion of polymers and the improvement of their interaction with the matrix, which is limited due the polymer hydrophobic nature, to decrease air entrapment in the fresh mixture;
- The rubber attached to polymeric and steel fibers from waste tires is another issue that requires deep study. It is true that it contributes to increase notably the impact energy absorption at first crack and at ultimate stage, but rubber particles reduce the fiber-matrix interactions, decreasing the compressive and flexural strengths compared to those of industrial steel FRC;
- The durability of hybrid R-FRC could be affected if the specific surface of recycled fibers is higher than that of industrial fibers. This is especially relevant in the case of recycled steel fibers because steel corrosion would be faster. On another hand, rubber attached to steel fibers would protect them from corrosion. Therefore, durability must be further studied in these cases;
- The interest and consolidation of 3D printing of concrete is increasing fast. Reinforcement of concrete structures made by 3D printing can be manually placed, for example, in form of steel bars. However, a better alternative is reinforcement by means of fiber dispersion, which need to be further explored [154]. In this sense, the use of hybrid recycled fiber reinforced SCC could be a good approach. However, the concrete for 3D printing must be easily pumped and its hydration rate must be fast enough to avoid the structure collapse. Therefore, the effect of fibers on workability and pumpability must be controlled without increasing the requirements of superplasticizer, which delay hardening.

In summary, the use of recycled fibers in hybrid R-FRC is a promising way to not only enhanced the product properties but also to improve sustainability and to reduce FRC costs. However, research is still need it to overcome the identified challenges related to fiber dispersion, quality and workability. Overcoming these challenges could open new application fields for hybrid R-FRC.

## Figures and Tables

**Table 1:** Properties of recycled fibers used in hybrid R-FRC.

	Length (mm)	Width (µm)	Apparent Density (kg/m^3^)	Melting Point (°C)	Young’s Modulus (GPa)	Tensile Strength (MPa)	Ref.
Steel (tire)	11	530	7850	1435	210	400	[70]
Steel (tire)	2–30	150	7850	-	210	2850	[82]
Steel (tire)	23	220		-	200	2570	[85,86,87]
Steel (tire)	20	150	7800	-	200	2850	[88]
Recycled polymer fiber (tire)	8.7	21.1	1160	>210	3.21	475	[86,87,89]
Copper (electrical conductors)	10	170	8760	-	-	387	[90]
Steel (galvanized binding wire)	20	800	7500	-	-	510	[90]
PP	47	700–1500	900–920	154–170	0.619	313	[84,91]
PP (carpets)	6	-	900		93.1–110	-	[92]
PE/PP (artificial turf)	10–40	330	985	-	-	-	[93]
PA (carpets)	5–11	38–41	-	258	5	286	[70]
PET (bottles)	4	500	1230	260	2.4	60	[59]
PET (embossed)	50	200	1380	-	10.2	420.7	[61]
PET	20–25	340	-	-	3.83	108	[94]
PET	40	2000–2500	1380	-	2.758	79.3	[95]
PET/PE (packaging)	10	800	1350	-	-	-	[93]
HDPE	3–10	100	-	-	0.672	25.22	[79]
Glass Fiber Reinforced Polymer	3.1–9.5	1.1–3.2	1760–2080	-	47.8–73.1	11.2–13.9	[96]

Nomenclature: HDPE: High Density Polyethylene; PA: Polyamide; PET: Polyethylene Terephthalate; PP: Polypropylene.

**Table 2:** Recycled metallic fibers (RMF) in hybrid R-FRC.

Cement Type	Fiber 1-(Dimensions: L, W, T or d)–Doses (in Volume Fraction)	Fiber 2- (Dimensions: L, W, T or d)–Doses (in Volume Fraction)	Recycling Source	Effect on Mechanical Properties	Other Effects	Application	Ref.
FRC: - OP 42.5 - w/c = 0.49 - Sand and coarse aggregates - Superplasticizer	Recycled steel fiber (L = 6–74 mm, d = 0.11–0.44 mm)—0.375%, 0.625% and 0.875%	Steel fiber (Non-recycled) (L = 6–70 mm, d = 0.15–1.20 mm)—0.375%	Recycled steel: waste tires	ΔCS ≈ 5–10% * ΔFirst crack strength ≈ 20% ** ΔDuctibility ≈ −3.8% ** (D_0_ index) and −14.5% ** (D_1_ index) (0.75% of mixed-total fibers, 50 recycled:50 non-recycled)	Recycled steel fibers turns the post-cracking behaviour of the FRC from crack-hardening to crack-plastic (reduction in the D_1_ index)	Structural applications	[37]
FRC: - OP 42.5 - w/c = 0.50 - Sand - Coarse aggregates - Superplasticizer	Recycled steel fiber (L = 9–15 mm, d = 0.11–1.64 mm)—0.5% (with 25%, 50% and 100% of non-recycled steel fibers replaced by an equal amount of recycled steel fibers)	Steel fiber (Non-recycled)—0.5%	Recycled steel: waste tires	ΔCS ≈ −6% ** ΔFirst crack strength ≈ 10.4% ** ΔDuctibility ≈ −28.2% ** (D_0_ index) and −9.2% ** (D_1_ index) (0.50% of mixed-total fibers, 50% of non-recycled steel fibers replaced by an equal amount of recycled steel fibers)	All R-FRC can be classified as “crack-softening” (both D_0_ and D_1_ < 1)	Structural applications	[106]
Rubberized FRC: - d = 0.5–2 mm - Replacing 5% vol of aggregates - Recycled rubber granules	Industrial steel fibers (L = 35 mm, d = 0.55 mm)—1.5% and 3% (w/v)	Recycled steel fibers (L < 15 mm, d = 0.18 mm)—1.5% and 3% (w/v)	Mechanical recycling of waste tires	Without rubber: ΔCS ≈ 0% ***, ΔT/L150 = 49% ** With rubber: ΔCS ≈- 20% ***, ΔT/L150 = 70% *** (50% industrial + 50% recycled steel fibers)	Cost savings up to 50% Impact strength increased up to 15% *** by using 5% rubber in FRC	Construction of high speed railways	[133]
FRC: - CEM II/ B-M SV 42.5N - w/c =0.46 - Crushed dolomite river sand - Superplasticiser	Recycled unshorted steel fiber (L = 0-15 mm (85% of the fibers), d = 0.55 mm)—3%, 5%, 8%, 12% and 15% (w/w)	Steel fiber (Non-recycled) (L = 35 mm, d = 0.55 mm)—1.7%, 2.4% (w/w)	Recycled steel: waste tires	ΔCS ≈ −1% ** ΔFS ≈ −4% ** (3% recycled + 1.7% industrial)	Does not affect workability (compared to industrial fibers) if dose of recycled fibers is less than 1.2% v	Structural applications	[38]
SCC: - OP 42.5R, - w/c =0.76 - Fly ash - Sand - Superplasticiser	Recycled steel fiber (L > 50 mm (63% of the fibers), d = 0.15 mm) –0.5–1%	Industrial steel fiber (non-recycled) (L/D = 47)—0.5–1%	Recycled steel: waste tires	ΔCS ≈ 50% * ΔTS ≈ 27%; ΔFS ≈ 35%* (0.5% recycled + 1% industrial fiber)	Impact strength increased up to 300% * (0.5% Recycled + 1% industrial fiber.)	Structural applications	[108]
SCC: - OP 42.5R, - w/c = 0.76 - Fly ash - Sand - Superplasticiser	Recycled steel fiber –0.15–1.35%	Steel fiber—0.15–1.35%	Recycled steel: waste tires	ΔCS ≈ 40–55% * ΔFS ≈ 25–40% * Both decreased with increasing recycled fiber fraction.	Impact strength increased up to 300% *. It decreased with increasing recycled fiber fraction	Structural applications	[108]
FRC: - OP - w/c = 0.55 - Coarse aggregate - Sand	Recycled steel fiber (L = 23 mm, d = 0.22 mm)—0.35%, 0.45% and 0.57%	Steel long fiber (LSF)(Non-recycled) (L = 60 mm, d = 1 mm)—0.35%, 0.45% and 0.57% Steel short fiber (SSF)(Non-recycled) (L = 55 mm, d = 0.8 mm)—0.35%, 0.45% and 0.57%	Recycled steel: post-processed steel fibers recovered from end-of-life tires	ΔCS ≈ 5% * ΔCS ≈ 20% ** ΔFS ≈ 70% * ΔFS ≈ 11% ** (0.28 recycled + 0.28 SSF) ΔCS ≈ −6% * ΔCS ≈ 1.6% ** ΔFS ≈ 20% * ΔFS ≈ −14% ** (0.28 recycled + 0.28 LSF)	Replaced of LSF or SSF by recycled fibers increased slump	Slabs-on grade and suspended slabs.	[85]
FRC: - OP - w/c = 0.55 - Coarse aggregate - Sand	Recycled steel short fibers (RSF) (L = 23 mm, d = 0.22 mm)—0.65%, 1%, 1.3 and 2% (w/w)	Recycled steel cord (RSC) (L = 60 mm, d = 0.75 mm)—0.65%, 1%, 1.3 and 2% (w/w)	RSC: un-vulcanised rubber belt off-cuts RSF: post-processed steel fibers from waste tires	ΔCS ≈ 22% * ΔCS ≈ 15% ** ΔFS ≈ 19% * ΔFS ≈ 13% ** (1% RSF + 1% RSC)	Deflection hardening behaviour Post-cracking strength increased 103% **	Concrete flooring applications	[135]
Reactive powder concrete: - w/c = 0.55 - Silica fume - Sand (0.6 mm) - Silica powder - Superplasticiser	Recycled steel fibers (L = 20–30 mm (47.6% of the fibers), d = 0.15–20 mm (40.9% of the fibers))—1%, 2%, 3% and 4%	Micro-steel fibers (Non-recycled) (L = 6 mm, d = 0.2 mm)—1%, 2%, 3% and 4%	Recycled steel: waste tires	ΔCS ≈ 25% *, ΔCS ≈ 20% ** ΔToughness = 200%*, 20% ***	Flowability decreases caused by fibers addition Better flowability with hybrid fibers than that for the non-hybrid mixture with the same amount of fibers	Structural applications	[80]
		Deformed steel fibers (Non-recycled) (L = 18 mm, d = 0.55 mm)—1%, 2%, 3%, 4% (v/v)					
FRC: - OP: IQS No.5 [136] - w/c = 0.39 - Sand - Superplasticizer	Recycled steel fiber (L = 20 mm, d = 800µm) —0.25–2.0%	Recycled copper fibers (L = 10 mm, d = 170 µm) —0.25–2.0%	Wastes from electrical connections and galvanized binding wires	ΔCS ≈ 20% *, ΔCS ≈ 77% *** ΔFS ≈ 105% *, ΔTS ≈140% * ΔFS ≈ −4% **, ΔTS ≈ −6% * (0.3% Coper + 0.7% Steel)	Flow table decreased 13% *	-	[90]
FRC: - OP 42.5R - w/c = 0.5 - Natural sand - Fine aggregate	Micro SSF (L = 10–16 mm, d = 200–300 µm)— 0%, 0.12%, 0.16%, 0.175%, 0.25%, 0.35% and 0.5%	HE (L = 30 mm, d = 650 µm)—0.12%, 0.16%, 0.175%, 0.25%, 0.35% and 0.5%	Scrap waste tires	ΔCS ≈ 0% *, ΔCS ≈ 0% ** ΔTS ≈ 13–28% * ΔRS ≈ 39% ** (0.175% SSF + 0.175% HE)	Enhancement of the resistance to abrasion (0.175% SSF + 0.175% HE; 0.25% SSF + 0.25% HE)	Repair mortar	[81]

Nomenclature: CS: Compressive Strength; d: diameter; FRC: Fiber Reinforced Composite; FS: Flexural Strength; HDPE: High Density Polyethylene; HE: Hook-end steel fibers; L: Length; LDPE: Low Density Polyethylene; OP: Ordinary Portland cement; RS: Residual Strength; SCC: Self-Consolidating Concrete; SSF: Scrap tire Steel Fiber; T: thickness; TS: Tensile Strength; W: Width; WFPRC: Waste Fiber and Powder Reinforced Concrete * compared to plain cement (without fibers); ** compared to single industrial fiber cement-based composite; *** compared to single recycled steel fiber FRC.

**Table 3:** Recycled synthetic polymeric fibers (RPF) in hybrid R-FRC from plastic bottle wastes.

Cement Type	Fiber 1- (Dimensions: L, W, T or d)—Doses (in Volume Fraction)	Fiber 2- (Dimensions: L, W, T or d)—Doses (in Volume Fraction)	Effect on Mechanical Properties	Other Effects	Application	Ref.
Lightweight wet-mix shotcrete (spray concrete): - OP 42.5 - w/c = 0.48 - Sand, natural gravel, walnut shell (as a replacement of natural gravel) - SF	Recycled PET (L = 20–25 mm, W= 2–3 mm, T = 0.34 mm)—0.045% (w/v)	PP (Non-recycled) —0.325% (w/v)	ΔTS ≈ 56% * (or 27% **) ΔCS ≈ 5% * (or 8% **)	ΔSlump ≈ −16% * ΔPdrop ≈ −20% * ΔRebound rate ≈ −15% * ΔBuilt-up thickness ≈ 25% *	Mine roof and mine roadways	[94]
SHCC: - OP - w/c = 0.48 - Class F fly ash - Sand (Silica or recycled sand)	Recycled PET fibers (L = 10 mm, d = 0.033 mm)—0.4%	PVA fibers (Non-recycled) (L = 12 mm, d = 0.039 mm)—1.6%	ΔCS ≈ 4.8% ** ΔFS ≈ −10.4% ** ΔTS ≈ −39.7% **	Environmental impact reduction ΔCO_2_ emissions = −0.5%**	Advanced construction material	[140]
SHCC: - OP 52.5 + calcium sulfoaluminate cement - Class F fly ash - Limestone powder - Silica sand (d: 120–212 μm) - Polycarboxylate-based superplasticizers	Untreated (U) and treated (T) recycled PET (rPET) fibers (L = 12 mm, d = 0.038 mm)—0.5%, 1%, 1.5% and 2%	PVA fibers (Non-recycled) (L = 12 mm, d = 0.039 mm)—0.5%, 1%, 1.5% and 2%	ΔCS ≈ −4.2 to 4.2% ** (for 28 days curing) and 11.2–29.6% ** (for accelerated curing) ΔTS ≈ −15, −30 and −44% **, (for 28 days curing); −26, −38 and −43% (for accelerated curing); for 25%, 50% and 75% of PVA replacement, respectively	Environmental impact and cost reduction ΔEmbody energy = −18.7% ** ΔCO_2_ emissions = −3.8% ** ΔCost = −39.9% ** (for 50% of PVA replacement)	Advanced construction material	[141]

Nomenclature: CS: Compressive Strength; d: diameter; FS: Flexural Strength; L: Length; OP: Ordinary Portland cement; PET: Polyethylene Terephthalate; PP: Polypropylene; PVA: Polyvinyl Alcohol; SHCC: Strain-Hardening Cementitious Composite; T: Thickness; TS: Tensile strength; W: Width. *compared to plain cement (without fibers); **compare to single fiber cement-based composite.

**Table 4:** Recycled synthetic polymeric fibers (RPF) in hybrid R-FRC from other recycling sources.

Cement Type	Fiber 1- (Dimensions: L, W, T or d)—Doses (in Volume Fraction)	Fiber 2- (Dimensions: L, W, T or d)—Doses (in Volume Fraction)	Recycling Source	Effect on Mechanical Properties	Other Effects	Application	Ref.
FRC: - CEM II B-S 32.5R - River sand of grains <2 mm - Butadiene-styrene resin with chalk filler	Recycled PA	Recycled PP	Recycled carpet	PA + PP fibers form a strong and water-resistant bond with concrete	PP or PA fibers has an insignificant impact on the wetting rate and the amount of imbibed water	-	[139]
CFRP concrete: - w/c = 0.45 - Melaminesulfonic acid agent (MA) (MA/c = 0.005) - Fine and coarse aggregate	Small and medium recycled and crushed CFRP (pieces made of epoxy reinforced with CF) (Small: L = 3.4 mm, d = 0.4 mm; Medium: L = 9.9 mm, d = 2.2 mm)—0.013%, 0.020% and 0.026%	Large recycled and crushed CFRP (pieces made of epoxy reinforced with CF) (L = 21 mm, d = 7.7 mm)—0.013%, 0.020% and 0.026%	CFRP	ΔFS ≈ 0–17%* (large-testing specimen = 100 × 100 × 400 mm) ΔCS ≈ −5–8.5%* (large-testing specimen = 100 × 200 mm) ΔWork of fracture (in the flexural test) ≈ 175–275%* (0.013–0.026% small size CFRP, respectively)	ΔSlump ≈ −54–90%* (0.013–0.026% small size CFRP, respectively)	Materials for repair and reinforcement buildings and infrastructures	[101]
Hybrid FRC beams: - Ordinary concrete - Natural aggregate and recycled aggregate (30% replacement)	Recycled PP fibers —0.038%, 0.075%, 0.113% and 0.151% (w/v)	Acrylic fiber —0.015%, 0.029%, 0.044% and 0.059% (w/v)	Textile waste	ΔCS ≈ 23.1%* ΔEM ≈ 28.2%* ΔTS ≈ 32.3%* (for 0.20% of mixed-fiber addition) ΔInitial cracking load ≈ 9.5% * (for 0.20% of mixed-fiber addition)	-	Structural components	[142]
Hybrid FRC beams: - OP 42.5 - w/c = 0.44 - Sand, natural aggregate and recycled aggregate (30% replacement) - Fly ash - Polycarboxylic acid superplasticizer	Recycled PP fibers (L = 19 mm)—0.038%, 0.075% and 0.113% (w/v)	PAN fibers (L = 19 mm)—0.133%, 0.089% and 0.044% (w/v)	PP: carpet	ΔCS ≈ 1.1% ** (for 25% of PAN replacement) ΔEM ≈ 5.3% ** (for 25% of PAN replacement) ΔTS ≈ 0.26% ** (for 75% of PAN replacement) ΔInitial cracking load of oblique section ≈ −20% ** (for 50% of PAN replacement)	-	Structural components	[143]

Nomenclature: CF: Carbon Fibers; CFRP: Carbon Fiber Reinforced Plastic; CS: Compressive Strength; d: diameter; EM: Elastic Modulus; FRC: Fiber Reinforced Composite; FS: Flexural Strength; L: Length; OP: Ordinary Portland cement; PA: Polyamide; PAN: Polyacrylonitrile; PP: Polypropylene; T: Thickness; TS: Tensile strength; W: Width. *compared to plain cement (without fibers); **compare to single fiber cement-based composite.

**Table 5:** Complex hybrid R-FRC containing recycled metallic materials and recycled plastics.

Cement Type	Fiber 1- (Dimensions: L, W, T or d)—Doses (in Volume Fraction)	Fiber 2 (Dimensions: L, W, T or d)—Doses (in Volume Fraction)	Recycling Source	Effect on Mechanical Properties	Other Effects	Application	Ref.
Hybrid FRC Beams: - PPC 53 Grade (IS1489) - w/c = 0.4 - Sand IS 4.75 - Crushed granite stones 10-20 mm - Superplasticiser	Scrim bled steel (Non-recycled) (L = 50 mm, d = 1 mm)—0.38%, 0.25% and 0.12%	Recycled PET (L = 38 mm, d = 0.02 mm)—0.38%, 0.25% and 0.12% PP (Non recycled) (L = 38 mm, d = 0.1 mm) —0.38%, 0.25% and 0.12%	-	ΔCS = 22% *, ΔTS = 17% * ΔFS = 19% *, ΔTS = 2% ** (0.38% steel fibers + 0.12% PET) ΔCS = 7.5% **, ΔTS = 12% **, ΔFS = 15% ** (0.38% steel fibers + 0.12% PP) 28 days curing	Increase in shear performance. First crack load increased 7% *	Structural applications	[145]
FRC: - C30/37 grade concrete -C55/67 grade concrete	Recycled steel (L = 25 mm)—4% (w/v)	PP (Non-recycled) (L = 54 mm)—0.45% (w/v)	Automotive industry waste steel fibers	ΔCS = −6% ** (for Concrete of 60 MPa) ΔCS = −11% ** (for concrete of 80 MPa)	Blast performance was kept	Protection of transport infrastructure against blast loading	[146]
Concrete: - OP ASTM type I - Crushed limestone 16 mm - Sand - Superplasticiser	Recycled metallic fibers (L = 30–60 mm)—1–3%	Recycled PP fibers (L = 30, 50, 60 mm)—0.5–1%	Metal—Locally available metal lathe workshop; PP—Storage bags	ΔCS = −20% *; ΔFS = 12% * 0.75% steel 60mm + 0.75% PP 60 mm	Improved post cracking behaviour	Normal concrete applications	[97]
FRC: - CEM I 42.5R - Sand - Coarse aggregate (river stones) - Rubber aggregate	Recycled steel and plastic fibers partially coated with rubber—8.5–42% (in weight/volume) replacing rubber aggregates. (L= 10–45 mm; d = 75% of them lower than 25 µm)		Waste tires	ΔCS = −36% *; ΔEM = −35% *, ΔFS = −7.3% *; ΔIEA1 = 100% *, ΔIEAU = 600% *;ΔCS = 9% ***; ΔEM = −3% ***; ΔFS = 15% *** (8.5% complex fibers) ΔIEA1 = 4500% * (33.5% complex fibers)	Bulk density increased 7% keeping similar thermal conductivity *** (8.5% complex fibers)	Conventional rubberized concrete for thermal rehabilitation of buildings	[51,52]
FRC: - CEM II/A-LL 42.5N w/c = 0.35 - Coarse aggregate - Fine aggregate - Sand - Superplasticiser	Metallic powders (mean size = 12 mm) – ~1%	Recycled PA fibers (L = 8 mm, d = 0.038 mm)—0.5%	PA – Textile carpet waste Rail steel	ΔCS = 18% *	Thermal conductivity increased due to steel 120–170% *	Thermal storage units for solar plants	[70]
	Recycled metallic shavings (L = 10–20 mm)—1%			ACS = −10% *			
SCC: - CEM I 42.5R - w/c = 0.6–0.64 - Fly ash - Silica fume - Superplasticiser	Micro-steel (L = 6 mm, d = 0.16 mm)—0.82% (w/v)	HDPE (L = 3–10 mm, d = 0.1 mm)—0.82% (w/v)	Municipal wastes	ACS = −30% * ACS = 0% ** AFS = −10% * AFS = 0% **	Drying shrinkage reduction: 11% * Flowing diameter reduction: 10% *	-	[79]
FRC: - CEM I 42.5R - Sand - Coarse aggregate - Rubber aggregate	Steel and textile fibers coated with rubber and rubber dust (7.7%—38.4% v/v) (replacing coarse aggregates 20—100%)		Waste from recycling rubber from waste tires	ΔCS = 9% *** (7.7% complex fiber with rubber)(28d)	Shrinkage 62% *** (7.7% complex fiber with rubber) Bulk density decreased	-	[52]
SCC: - OP 42.5R - w/c = 0.76 - Fly ash - Aggregate - Superplasticizer	Recycled steel fiber (L = 50 mm, d = 0.15 ± 0.5 mm) 0.5%, 0.75% and 1%	PP (Non-recycled) (aspect ratio = 461) 0.5%, 0.75% and 1%	Recycled steel: waste tires	ΔCS = 30% *; ΔCS = −12% ** ΔFS = 20% *; ΔFS = −10% ** (0.5%PP + 1% recycled steel)	Impact energy absorption increased 1800% * (0.5%PP + 1% recycled steel)	-	[108]
- OP IS 12269 - w/c= 0.4 - Sand IS 4.75 - Crushed granite stones 10–20 mm - Silica fume - Superplasticiser	Recycled steel fibers (L = 10–15 mm)	Recycled nylon fibers (L = 40 mm)	Steel lathe waste Nylon waste from local industries	Best performance: total fiber = 2% ΔCS = 12% *; ΔTS = 54% * (steel/nylon ratio = 2) ΔMOR = 50% * ΔIE1 = 238% *; ΔIEU = 205% *; ΔFE = 197% * (steel/nylon ratio = 1)	Air content increased up 75%	-	[147,148]
	Total fiber fraction 0.5%, 1.0%, 1.5% and 2.0% Steel/nylon ratios: 1/2, 1, 2 Superplasticiser at demand to get desired workability						
SCC: - CEM I 42.5 R -w/c = 0.38 - Fine aggregates -superplasticiser	Recycled steel fiber (L = 50 mm, d = 0.15 mm)—0.35%, 0.7% and 1.05% (in volume fraction)	PP (Non-recycled) (L = 12 mm, d = 0.018 mm)—0.35% and 0.7% (in volume fraction)	Recycled steel: waste tires	Best performance: ΔCS = 39% *; ΔFS = 31% * ΔCS = 11% **; ΔFS = 10% ** (1.05% steel + 0.7% PP) ΔIE1 = 27% * (0.7% steel + 0.7% PP)	Slump flow diameter decreased linearly with fiber fraction ΔSlump = −19% * (1.05% steel + 0.7% PP)	-	[144]
FRC: - CEM I 52.5N - w/c = 0.4 - Sand - Crushed granites - Superplasticiser	Recycled steel fiber (L = 23 mm, d = 0.22 mm)—0.5–0.9%	PP (Non-recycled) (L = 12 mm, d = 0.05 mm)—0.1–0.5%	Recycled steel: waste tires	Best performance: ΔCS = −5% *; ΔFS = −1% * ΔCS = −10% **; ΔFS = −14% ** ΔTS = 40% *; ΔTS = −34% ** (0.9% steel + 0.1% PP)	Increase chemical resistance in chlorine environments Decrease shrinkage 5% **–35% ** (0.4%PP + 0.6% steel)	-	[89]
- CEM II 52.5 - w/c = 0.56 - Coarse aggregate - Fine aggregate - Superplasticizer	Recycled steel fibers (L = 19–21 mm, d= 0.11–0.19 mm) 40 kg/m^3^	Recycled polymer fibers (L = 4–30 mm, d= 0.02–0.03 mm) 2 kg/m^3^ and 5 kg/m^3^	Waste tires	ΔCS = −1% * (40 kg/m^3^ steel + 5 kg/m^3^ polymer)	Prevention of fire spalling	FRC-lined tunnels	[149]

Nomenclature: CS: Compressive Strength; d: diameter; EM. Elastic Modulus; FRC: Fiber Reinforced Composite; FS: Flexural Strength; HDPE: High Density Polyethylene; L: Length; OP: Ordinary Portland cement; PPC: Portland Pozzolana cement; SCC: Self-Consolidating Concrete; T: Thickness; TS: Tensile Strength; W: Width; IEA1: Impact Energy Absorption at first crack; IEAU: Impact Energy Absorption at ultimate stage. * compared to plain cement (without fibers); ** compared to single recycled fiber cement-based composite, *** compared to the equivalent rubberized concrete (with high quality recycled rubber) without fibers.

**Table 6:** Complex hybrid R-FRC containing other recycled material.

Cement Type	Fiber 1- (Dimensions: L, W, T or d)—Doses (in Volume Fraction)	Fiber 2 (Dimensions: L, W, T or d)—Doses (in Volume Fraction)	Recycling Source	Effect on Mechanical Properties	Other Effects	Application	Ref.
SCC: - CEM I 42.5 R - w/c = 0.35–0.38 - Sand - Coarse aggregates	Recycled steel fibers (L = 8 mm, d = 0.175 mm) + Recycled steel fibers (L = ~2–30 mm, d = 0.15 mm)—1.5%	Glass fibers (L = 12 mm, d = 0.014 mm)—0.5%	Recycled steel: waste tires	ΔCS = 2% *	Increase energy absorption up to 30% *	Building structures	[82]
- OP 42.5 R - w/c = 0.5 - Fly ash - Sand - Crushed waste concrete - Superplasticiser	Recycled PP (L = 6 mm)— 0–0.3%	Basalt fibers (L = 18 mm)— 0—0.3%	Used carpets	ΔCS = −25% ** ΔTS = −20% ** ΔFS = 10% ** (0.15% of each fiber)	Workability did not change with the hybridaton	-	[92]
- CEM I 52.5 N - w/c =0.21 - Silica fume - Sand - Superplasticiser	Recycled fibers (glass + unsaturated polyester) (L = 0.4–23 mm)—4.41% and 6.2%	Recycled powder (glass + unsaturated polyester) (L = 0.1–0.4 mm)—7.13%	Thermoset composite	ΔTS = 80% * (4.41% hybrid fibers 7.13% powder) ΔTS = 54% * (4.41% hybrid fibers)	Slump flow decreased −40% * when fibers and powder were used	-	[56]
- OP - w/c = 0.33	Recycled carbon	Recycled glass	Polymer core composite conductors	ΔCS = −25% *	ΔHardness = 11% * Increase resistance to sea water		[57]
	Particle fiber powder 6 wt.%						

Nomenclature: CS: Compressive Strength; d: diameter; FS: Flexural Strength; L: Length; OP: Ordinary Portland cement; SCC: Self-Consolidating Concrete; PP: Polypropylene; TS: Tensile Strength. * compared to plain cement (without fibers); ** compared to single recycled fiber cement-based composite, *** compared to the equivalent rubberized concrete (with high quality recycled rubber) without fibers.

## Data Availability

Data sharing not applicable.

## Abbreviation

CF	carbon fibers
CFRP	carbon fiber reinforced plastic
CS	compressive strength
d	diameter
EM	elastic modulus
EPS	expanded polystyrene foam
FRC	fiber reinforced composites
FS	flexural strength
HDPE	high density polyethylene
HE	hook-end steel fibers
IEA1	impact energy absorption at first crack
IEAU	impact energy absorption at ultimate stage
L	length
LCA	life cycle assessment
LDPE	low density polyethylene
LWC	lightweight concrete
OP	ordinary Portland cement
PA	polyamide
PAN	polyacrylonitrile
PET	polyethylene terephthalate
PP	polypropylene
PPC	Portland pozzolana cement
PVA	polyvinyl alcohol
PVC	polyvinyl chloride
R-FRC	recycled fiber reinforced composites
RGF	recycled glass fibers
RMF	recycled metallic fibers
RPF	recycled synthetic polymeric fibers
RS	residual strength
RTSF	recycled-tire steel fibers
SBF	sugar beet fiber
SCC	self-consolidating concrete
SHCC	strain-hardening cementitious composite
SSF	scrap tire steel fiber
T	thickness
TS	tensile strength
W	width
WFPRC	waste fiber and powder reinforced concrete
